# Haemoglobin status to determine nutritional anaemia and its association with breakfast skipping and BMI among nursing undergraduates of Farasan Island, KSA

**DOI:** 10.1017/jns.2022.33

**Published:** 2022-05-24

**Authors:** Shabihul Fatma Sayed, Sumathi Nagarajan

**Affiliations:** College of Nursing, Farasan Province, Jazan University, Jazan, Kingdom of Saudi Arabia

**Keywords:** Body mass index (BMI), Breakfast skipping, Hb content, Nutritional anaemia

## Abstract

The present study was conducted to determine nutritional anaemia using haemoglobin levels of female nursing undergraduates studying at Farasan Island with the purpose to intervene at a point, before the potential problems become serious later in life. In total, 130 apparently healthy, female students of Department of Nursing were recruited by a random sampling method to collect information on socio-demographic, lifestyle and anthropogenic characteristics, and dietary habits including breakfast skipping. Haemoglobin content was estimated using Sahli's Haemoglobinometer and observations were interpreted as per WHO's criteria for anaemia. Body mass index (BMI) was recorded using a digital weighing machine. Correlation between haemoglobin concentration, breakfast skipping and body mass index of study participants was assessed by Pearson's correlation. Data analyses were done using Origin software. Overall, 51⋅6 % (*n* = 67) students were all together anaemic with 28⋅5 % (*n* = 37) had mild anaemia, 15⋅4 % (*n* = 20) moderate and 7⋅69 % (*n* = 10) had severe anaemia. Of these, 20⋅8 % (*n* = 27) were underweight, 63⋅8 % (*n* = 83) normal weight and 15⋅4 % (*n* = 20) were above normal weight (over weight and obese). The Hb content showed a positive correlation with the BMI and exhibited an increasing trend with increase in the BMI among study participants (*P* < 0⋅05). Questionnaire analyses revealed that the majority (96⋅9 %, *n* = 126) of students were taking junk food as bulk of their meal. A strong negative correlation was recorded between Hb contents and breakfast skipping tendencies (*r* = −0⋅987, *P* < 0⋅05). Findings of the present study are of high significance for public health professionals and educators to prioritise actions that could motivate these future nurses to adapt healthy lifestyles to strategically combat nutritional anaemia.

## Introduction

Anaemia is a public health issue that affects low-, middle- and high-income countries and has adverse impacts on socio-economic development. Low oxygenation of brain tissues due to anaemia may lead to impaired cognitive functions and psychomotor development, especially among children and teens. It adversely affects learning, cognitive function, behaviour, attention and regular activities of young students^(1–3)^ and may also results in college absenteeism. Anaemia has multifactorial aetiology such as nutrition, age, sex, social class, lifestyle characteristics, anthropogenic factors which are reflection of obesity such as BMI, dietary habits and infections. Nutritional anaemia is one of the most important global health issues and is the most common morbidity among micronutrients and affects health, education, economy and productivity of the entire nation^(4)^. It is categorised as one of the ten most serious health problems by the World Health Organization^(5)^ and is defined by the WHO as Hb<13⋅0 g/dl in male adults and <12⋅0 g/dl in female adults^(6)^. In 2016, the global prevalence of anaemia among women of reproductive age was 32⋅8 % (compared with 30⋅3 % in 2012). The rates of anaemia were highest in South-East Asia (45⋅8 %), Eastern Mediterranean (39⋅8 %) and African (39⋅0 %) regions^(7)^.

The term ‘nutritional anaemia’ encompasses all pathological conditions in which the blood haemoglobin concentration drops to an abnormally low level, due to a deficiency in one or several nutrients. Its adverse health consequences affect people of all age-groups^(8)^. Complete and balanced nutrition is essential to maintain normal health and avoid health risks throughout the entire life. Moreover, during the development stage and, particularly, during early adolescence, good nutrition is essential as it influences brain maturation and has impact on future health indicators^(9,10)^. Increased activity, social life and the busy academic schedules of university undergraduates may affect their eating habits. Females in their teenage have higher nutrient requirements due to their rapid growth, expansion of red blood cells, dramatic psychological changes, loss of blood during menstruation, increased physical activities which are the characteristics of adolescence, and therefore are at higher risks of anaemia incidence if their nutritional needs are not met. If this happens for a long time, it will cause Hb levels continue to decrease and cause anaemia^(11)^.

Breakfast is the most essential meal of the day^(12)^. Many previous studies have exhibited the proven positive impact of breakfast in appetite regulation^(13)^. Although the variety of factors affect a person's nutritional status, irregular food consumption pattern mainly the breakfast skipping tendencies are the most dominant factor that makes young university females vulnerable to the development of anaemia because continued breakfast skipping makes the body unable to meet the diversity of nutrients needed for the synthesis of haemoglobin (Hb). Despite all these facts, breakfast is frequently skipped, especially among the young generation, with almost 74 % of female students in Saudi Arabia either skip or irregularly consume their breakfast^(12,14)^.

Furthermore, high overweight and obesity prevalence has been observed in developed and developing countries, and obesity is considered as an important public health problem worldwide^(15,16)^, mainly due to its close relationship between inadequate nutritional status leading to various serious health issues including nutritional anaemia. Overweight is multifactorial in origin, with important genetic^(17)^ and environmental factors such as inadequate eating habits, the preference for quick meals, consisting mostly the high-calorie foods like snacks and soft drinks^(18)^. It may be considered that anaemia holds a close association with body type and various anthropometric parameters^(19–27)^. Thus, anthropometric parameters can serve as alternative predictors of anaemia, which are simple, non-invasive and rapid yet accurate. The most studied anthropometric parameters that find an association with haemoglobin levels include body mass index^(28–30)^ which gives significant information on the nutritional and health status of individuals. For this reason, BMI has been considered as one of the parameters to determine nutritional status of the study participants and hence nutritional anaemia. The majority of studies have associated anaemia with low BMI^(31–34)^. Although various studies have been conducted in the past on Hb content anaemia and the associated risk factors at the national^(35–41)^ and international levels^(30,34,42–45)^, information of anaemia and its association with breakfast skipping, and BMI is not available. To the best of our knowledge, based on PubMed, Scopus and Google scholar database search, this is the first study that reports anaemia and its association with breakfast skipping, and BMI.

Farasan is a beautiful Island located about 40 km offshore from the city of Jazan. It is close to being registered under UNESCO's Man and Biosphere Program^(46,47)^. It has a vast educational institute, as Farasan University College with well-established Department of Nursing, affiliated to Jazan University. Despite all its beauty and natural wealth, scientifically it has been less studied. Farasan is famous for its coral reefs, pristine beaches, crystal clear waters and, rich land and underwater wildlife, and for its Parrot-fish locally called as ‘hareed’. Considering the increasing trend of breakfast skipping^(12,14,48)^, obesity/overweight in KSA^(49)^ and the higher prevalence and public health outcomes of anaemia^(50)^, the study on the association of anaemia with breakfast skipping and BMI could help meet the challenges of anaemia and complications related to breakfast skipping due to malfunction in dietary intake. Since young girls of age 18–25 years are more prone to get nutritional anaemia as this age group is vulnerable to dietary deficiencies because of their increased physiological needs of micronutrients, it is crucial to expand our understanding on nutritional anaemia and its association with breakfast skipping, and BMI. The outcomes would be of high significance for public health professionals and educators to prioritise actions that could motivate these future nurses to adapt healthy lifestyles to combat nutritional anaemia.

## Materials and methods

### Study design

A cross-sectional study was conducted to determine nutritional anaemia among the nursing undergraduates. The study was conducted from the beginning of November 2020 to April 2021. Anaemia was determined by analysing blood samples to measure the concentration of haemoglobin (Hb g/dL). The correlation of Hb contents with breakfast skipping and body mass index (BMI) was assessed. Dietary habits for breakfast skipping of study participants were evaluated by questionnaire analyses.

### Study subjects

The study included 130 eligible nursing undergraduates (18–25 years) from Department of Nursing at Farasan University College, Jazan University, Kingdom of Saudi Arabia. Study participants were randomly selected.

### Ethical statement

This study was conducted according to the ethical guidelines of Jazan University and was approved by the College Ethics Committee (Farasan/2020-1). The study protocol strictly followed the Declaration of Helsinki. Participants were informed about the study objectives and enrolled after getting their voluntary consent, researchers conducted a face-to-face interview with each student during their scheduled intervals. Informed consent forms, along with a self-administered questionnaire, were sent to the WhatsApp group of students. Each student was given the complete unconditional choice to participate without any incentives/bonus or penalty and was assured that confidentiality of data throughout the study would be maintained and that the data would be used exclusively for research.

### Inclusion criteria

Apparently healthy participants who gave their voluntary consent and were full-time students enrolled with Jazan University during the academic year 2020/2021 were included. Information pertaining to this has been summarised in Table 1.

**Table 1.** Demographic, lifestyle and anthropogenic characteristics, and the dietary habits of study participants (*N* = 130)

Variables	Frequencies	%	*P*-value*
*Section A: Socio-demographic characteristics*			
Mean age (22 ± 3 years)			
≤20 years	45	34⋅62	0⋅04
>20 years	85	65⋅38	
Sex			
Male	None	None	
Female	130	100	
Siblings			0⋅16
Between 1–4	81	62⋅31	
>4	49	37⋅69	
Nationality			
Saudi	130	100	
Non-Saudi	None	–	
Family's Educational Background			0⋅49
Educated	130	100	
Uneducated	None	00	
Father's education			0⋅55
Primary	None	–	
Secondary	43	33⋅1	
Graduate and above	87	66⋅9	
Mother's education			0⋅21
Primary	44	33⋅9	
Secondary	61	46⋅9	
Graduate and above	25	19⋅2	
Social status			0⋅09
Lower-middle class	–	–	
Middle class	–	–	
Upper-middle class	89	68⋅5	
Affluent	41	31⋅5	
Marital status			
Single	130	100	–
Married	None	00	
Residence			
Farasan	127	97⋅7	
Outside Farasan	03	2⋅3	
Accomodation			
Hostelers (living in rented apartments)	15	11⋅54	0⋅03
Dayscholars	115	88⋅5	
Daily travellers	–	–	
Study Year	2020–2021		0⋅04
1st	26	20⋅0	
2nd	25	19⋅2	
3rd	57	43⋅9	
4th	22	16⋅9	
*Section B: Lifestyle characteristics*			
Lifestyle			
Active	125	96⋅2	0⋅04
Sedantary	05	03⋅8	
Do you follow good hygiene practices			
Yes	130	100	
No	00	00	
Practice handwashing before eating			
Yes	127	97⋅7	
No	3	2⋅3	
Menstruation cycle			
Regular	124	95⋅4	0⋅01
Irregular	06	04⋅6	
Duration of Menstruation cycle			
<7 d	122	93⋅9	0⋅02
≥7 d	08	06⋅1	
Smoke			
Yes	00	00	
No	130	100	
Any previous surgery			
Yes	00	00	
No	130	100	
Family history of chronic disease (specifically diabetes)			
Yes	00	00	
No	130	100	
Blood transfusion			
Yes	00	00	
No	130	100	
Body weight			
Normal	83	63⋅8	0⋅03
Underweight	27	20⋅8	
Overweight	11	8⋅5	
Obese	09	6⋅9	
Height			
Short	07	5⋅40	
Average	105	80⋅7	
Tall	18	13⋅9	
*Section C: Dietary habits*			
Prefer junk foods			
Yes	126	96⋅9	0⋅01
No	04	3⋅1	
Soft drinks			
Once daily	110	84⋅6	
Twice daily	20	15⋅4	
Thrice daily	00	00	
Not at all	00	00	
Hot drinks			
Black tea	130	100	0⋅01
Kahwa	130	100	
Turkish coffee	100	76⋅9	
Black coffee	100	76⋅9	
Food preference			
Vegetarian	00	00	0⋅55
Non-vegetarian	130	100	
Breakfast			
Regular	30	23⋅1	0⋅03
Skip (three or more times/week)	100	76⋅9	
If skip, reasons for breakfast skipping			
Lack of time	100	76⋅9	0⋅04
Lack of interest in breakfast	00	00	
Unavailability of breakfast	00	00	
Dairy products (milk)			
Yes	126	96⋅9	0⋅03
No	04	3⋅1	
If yes, then which category			0⋅04
Camel milk	14	10⋅8	
Cow milk	104	80⋅0	
Goat milk	08	6⋅2	
Take green leafy vegetables			
Once in a week	00	00	
Twice in a week	00	00	
Thrice in a week	130	130	
Not at all	–	–	
Take fresh fruits			
Once in a week	–	–	
Twice in a week	–	–	
Thrice in a week	100	76⋅9	
Not at all	30	23⋅1	
Prefer Camel meat			
Yes	130	100	
No	00	00	
Prefer seafoods			
Yes	130	130	0⋅02
No	00	00	
Taking any iron supplement			
Yes	23	–	
No	107	–	

* *P* < 0⋅05 is statistically significant.

### Exclusion criteria

Students on routine medications due to any type of medical conditions were not included in the study. Those who were having their menstruation during sampling were also not included. Students with bleeding disorders or with the history of haematological disorders and those who had undergone minor or major surgery in recent past were also excluded from the study.

### Data collection

A self-structured questionnaire was developed after a comprehensive review of relevant literatures published in peer-reviewed journals only^(51–57)^. The questionnaire had three domains: Section A was dedicated to general Demographic information which included twelve items on age, gender, educational background, nationality, marital status, and study year. Section B had eleven items related to lifestyles and anthropometric characteristics and Section C had thirteen items which were exclusively focused on dietary habits of the study participants which included intake of regular breakfast, frequency of junk food intake, multivitamins and the questions on intake of iron/iron-rich foods (Table 1).

Data collection took nearly 4 months. All sampling and data collections were done under strict guidelines of COVID-19 safety protocol which may be the reasons that data collection took more time.

### Estimation of Hb content

To collect data on Hb content of the participants, blood samples were drawn by Sahli's pipette and added to the haemoglobin tube where haemoglobin (Hb) was converted to acid haematin by the addition of 0⋅1 N HCl. The resulting brown colour was diluted by distilled water to finally match with the standard brown glass reference blocks of Sahli's haemoglobinometer^(58)^. Results were noted as g/dl. The measured values were tabulated and compared to the standard values of grading anaemia according to the WHO guidelines with <12 g/dL of haemoglobin considered as anaemic^(5)^, while values of haemoglobin of 10⋅0–11⋅9 g/dL, 7⋅0–9⋅9 g/dL and <7 g/dL were noted as Grade 1 (mild) anaemia, Grade 2 (moderate) anaemia and Grade 3 (severe) anaemia, respectively. Good laboratory practice and quality control were maintained while blood sampling.

### Anthropometric measurements

Anthropometric measurements related to weight and heights were taken by the researcher. Body weight and BMI were measured by a self-calibrating digital weighing machine in standardised manner with the students standing bare feet and wearing light clothes (Seca, Digital, Germany). Height measurements were reassured by using a stadiometer (Detecto, Patriot, USA) measured to the nearest 0⋅1 cm. Anthropometric measurements were used to get BMI data.

A value of BMI <18⋅5 kg/m^2^ was considered as underweight, 18⋅5–24⋅9 kg/m^2^ as normal weight, 25⋅0–29⋅9 kg/m^2^ as pre-obese (overweight) and BMI 30 kg/m^2^ was considered as obese^(16,59)^.

### Breakfast skipping

Breakfast skipping was calculated by the evaluation of questionnaire response. Breakfast is generally considered as the foremost meal taken after waking up in the morning between 5:00 am and 11:00 am^(60,61)^. Breakfast skipping is defined as not taking breakfast one day or more per week^(62,63)^. The breakfast skipping was measured by asking the participants about the number of days per week they usually did not take the breakfast. The responses for reasons of skipping the breakfast were also calculated as frequencies and percentages.

### Statistical analysis

Data were collected and analysed using Origin (version 8⋅1, Originlab.com, USA). Descriptive statistics were conducted where quantitative data was shown as mean and standard deviation (sd). The qualitative data were expressed as frequency and percentage. Inferential statistics was performed as *χ*^2^ test to measure association between qualitative variables, while independent Student's *t* test was used to compare mean and sd of two sets of quantitative normally distributed data. Pearson's correlation was used to determine the type of correlation between Hb content (g/dL), breakfast skipping and BMI of the study participants. *P*-value was considered statistically significant at *P* < 0⋅05.

## Results

Socio-demographic and health characteristic data on study participants are presented in Tables 1 and 2. The questionnaire analyses revealed that all the participants belonged to upper-middle class or affluent class (Table 1). Family's educational background and the study year of the participants had no significant relation to anaemia (*P* > 0⋅05, Table 1). Regarding lifestyle characteristics data, 96⋅2 % of the study participants reported that they have an active lifestyle and follow a scheduled evening walk and exercising activities in college gym during their free lecture timings.

**Table 2.** Descriptive statistics of study population

Variables	Mean* (*n* = 130)	sd*
Age	22⋅1	3⋅31
Height in cm	155⋅33	2⋅56
Weight in kg	49⋅59	9⋅15
Body mass index (BMI)	21⋅68	3⋅67
Haemoglobin in g/dl	11⋅47	1⋅57

* Continuous variables were analysed using an independent samples *t* test and are expressed as means and standard deviations.

### Hb contents of study participants

The mean Hb g/dL of the study population was 11⋅5 g/dL (Table 2).

### Anaemia characteristics of study participants

Anaemia was present in 51⋅6 % (*n* = 67) of the study participants with 28⋅5 % (*n* = 37) had mild, 15⋅4 % (*n* = 20) moderate and 7⋅69 % (*n* = 10) had severe anaemia (Table 3).

**Table 3.** Distribution of the study sample according to the grade of anaemia

Haemoglobin (g/dL)	Indicator	Frequency	Percentage	Hb (g/dL)	sd	*P*-values*
≥12	Non-anaemic	63	48⋅46	12⋅7	0⋅4	0⋅04
10⋅0–11⋅9	Grade 1 (mild) anaemia	37	28⋅5	11⋅7	0⋅1	0⋅01
7⋅0–9⋅9	Grade 2 (moderate) anaemia	20	15⋅4	9⋅1	0⋅2	
Less than 7	Grade 3 (severe) anaemia	10	7⋅69	6⋅5	0⋅3	

* Statistically significant at *P* < 0⋅05. Categorical variables were analysed using *χ*^2^ test and expressed as numbers and percentages. Continuous variables were analysed using an independent samples *t* test and are expressed as means and standard deviations.

### Breakfast skipping

Breakfast skipping was calculated by the evaluation of questionnaire response. Based on questionnaire analyses, it was found that only 23⋅1 % (*n* = 30) participants had daily breakfast habits, while 76⋅9 % (*n* = 100 students) skip breakfast regularly (Table 4).

**Table 4.** Reasons of breakfast skipping

Reasons of breakfast skipping	Frequency of study participants	Percentage of study participants	*P*-values*
Lack of time	100	76⋅9	0⋅03
Lack of interest in breakfast	00	00	–
Unavailability of breakfast	00	00	–

* Categorical variables were analysed using *χ*^2^ test. Statistically significant at *P* < 0⋅05.

The study participants who regularly skip breakfast had significantly lower mean haemoglobin status (11⋅5 g/dL, *r* = −0⋅983, *P* < 0⋅05) and hence were more likely to get anaemic compared to those who were regular in their breakfast (12⋅4 g/dL), although the number of such students were quite less. This clearly indicates that nutrients responsible to tackle anaemia were not taken in sufficient amount by the participants who were skipping breakfast and hence were anaemic. Those who regularly skip the breakfast cited the reasons as lack of time.

### Anthropogenic measurements as BMI assessments

As exhibited by data of BMI, 20⋅8 % (*n* = 27) study participants were found to be under weight. Irrespective of their anaemic status, the majority of study subjects regularly skip breakfast and frequently consume junk foods as their diet and hence were at risk of developing anaemia.

Data pertaining to BMI of study participants exhibited that the frequency of anaemia was more among those who were underweight and was significantly less (*P* < 0⋅05) among overweight participants compared to the other study groups (Table 5). Furthermore, Hb content and BMI of the study participants exhibited a significant positive correlation (*r* = 0⋅187, *P* < 0⋅05, Table 6). It was noted that underweight students had the lowest mean haemoglobin content (6⋅2, sd 0⋅8 g/dL) and were severely anaemic. Moreover, overweight and obese students had highest mean haemoglobin concentration (12⋅5, sd 0⋅6 g/dl).

**Table 5.** Distribution of the study sample according to body mass index (BMI)

Body mass index (BMI)	Frequency	Percentage	*P*-value*
Normal	83	63⋅8	0⋅03
Underweight	27	20⋅8	
Overweight	11	8⋅50	
Obese	09	6⋅90	

* Categorical variables were analysed using *χ*^2^ test. Statistically significant at *P* < 0⋅05.

**Table 6.** Association of breakfast skipping and body mass index (BMI) with Hb content

Blood haemoglobin	Significance level	Breakfast skipping	Body mass index (BMI)
Hb (g/dL)	*r*	−0⋅983	0⋅187
	*P*	0⋅01	0⋅03

*r* = Pearson's correlation.

*P* = Statistical significance at *P* < 0⋅05.

## Discussion

Anaemia remains a major public health problem, affecting one third of all adults and almost two billion people worldwide^(64)^. Defined broadly as a condition associated with lower than normal haemoglobin concentration, anaemia impairs the circulation of oxygen in the blood, which in turn has detrimental effects on maternal and birth outcomes, suboptimal child growth, impaired learning, reduced work productivity and income earning during adulthood^(65)^.

Despite the fact that the participants of the present study belonged to upper-middle class or affluent class, significant percentages of participants (51⋅6 %, *n* = 67) were found to be anaemic indicating that the incidence of nutritional anaemia is not exclusively related to poverty alone. Various other studies have also reported insignificant association between the prevalence of anaemia and socio-economic class^(36,66)^. The majority of study participants of the present study had mild anaemia which is also seen in other studies^(67)^. Despite being well educated and well oriented about nutrition and its ill effect on health, anaemia is prevalent among nursing undergraduate students. BMI exhibits a higher prevalence of anaemia among underweight followed by normal weight and overweight participants. The prevalence of anaemia among 51⋅6 % study participants is a serious health issue as per WHO's classification^(5)^. Findings of the present study are similar to various other studies, done on university students both inside the Kingdom and various international studies^(38,67,68)^.

Though we did not encounter more cases of severe anaemia in the present study, however, 15⋅4 % (*n* = 20) and 28⋅5 % (*n* = 27) of moderate and mild anaemia cases in the present study are worrisome findings. This study population is ideally supposed to have a better awareness and access to anaemia diagnosis and treatment compared to general population as all are the future healthcare professionals. These findings are similar to the study conducted by Pandey and Singh^(69)^ among the medical students, where they found that there was mild anaemia among twenty students (20⋅83 %) followed by moderate anaemia among nine students (9⋅37 %), however in another study by Keller^(70)^ severe cases of anaemia was not found among the study participants. The findings are also comparable to a study conducted by Chaudhary *et al.*^(71)^, where out of 104 subjects, 72 subjects (69⋅2 %) had mild anaemia while 32 subjects (30⋅8 %) had moderate anaemia and none of their study subjects had severe anaemia^(1)^.

Although many factors affect a person's nutritional status, adequate nutrition is the most dominant factor for normal nutritional status^(72)^. This is consistent with research conducted by Shariff and Akbar^(73)^ which states that there is an influence between the levels of nutritional consumption on anaemic status of female students. Various other factors that trigger the occurrence of nutritional anaemia among adolescence are the wrong eating habits, wrong understanding of nutrition to attain a slim body, and also excessive preference for certain foods is prevalent among teenagers hindering their nutritional needs.

Anaemia is directly affected by the consumption of daily foods^(73)^. Despite the documented benefits of breakfast consumption, skipping breakfast was found to be more common and the percentage of participants skipping regular breakfast was 76⋅9 %. It has also been reported in various other studies that the frequency of skipping breakfast is higher in girls^(74,75)^, and breakfast skippers tend to consume more fast food, leading to increased weight gain from adolescence to adulthood. These unhealthy dietary habits are also seen in various other such studies done in the past^(76–78)^. Evidence suggests that regular breakfast intake improves the cognitive function and have positive impact on the health of the children and young adults^(79,80)^. Since breakfast is the first meal of the day which sets the metabolic rate, if breakfast is skipped, brain interprets it as starving setting a lower metabolic rate leading to weight gain when one eats later. The habit of breakfast skipping is also a risk factor for the incidence of anaemia among educated youth^(81,82)^. Research conducted by Ansar *et al.*^(83)^ found that young women who did not eat breakfast showed lower mean haemoglobin (12⋅03 g/dl) compared to those who were regular in their breakfast (12⋅63 g/dl). Based on the results of the study, it can be concluded that breakfast habits have significant influence on the incidence of anaemia.

A significantly low BMI in women indicates disorder and malnutrition. Overall, a BMI level at 18⋅5 kg/m^2^ was more common among the study participants. Although in the present study, the relatively low frequency of overweight and obesity was recorded, and hence, BMI above 30 kg/m^2^ was least common. Analysis by BMI categories in the present study showed that the percentage of anaemia was found to be high among underweight students (88⋅9 %) as compared to normal (45⋅8 %), overweight (27⋅3 %) and obese participants (11⋅1 %). Studies conducted by various other scientists have also documented almost similar findings^(10,32–34,80)^. These findings suggest that good nutritional status reduces the risk of anaemia. A large prevalence of anaemia is attributed to nutritionally inadequate diet among the females^(80)^. Pandey and Singh^(69)^ found the prevalence of anaemia among underweight, normal weight and overweight was 60, 27⋅5 and 12⋅5 %, respectively. Gupta *et al.*^(84)^ found the higher prevalence of anaemia among underweight 91⋅4 % followed by normal weight 83⋅6 % and in overweight participants 73⋅3 %. Similarly, Pal *et al.*^(85)^ found the higher prevalence of anaemia among underweight males and females as 62⋅5 and 80⋅65 %, respectively, among normal weight males and females 45⋅98 and 62⋅67 %, and among overweight/obese males and females 19⋅05 and 25⋅0 %, respectively. Waseem *et al.*^(86)^ found 44⋅9 % anaemia among underweight participants, 23⋅67 % among normal weight while 10 % anaemia was recorded among overweight participants. Furthermore, Sinha *et al.*^(24)^ reported the prevalence of anaemia among undernourished women at 76⋅06 % compared to those with normal weight and overweight women, where the prevalence was at 75⋅28 and 66⋅67 % indicating a positive correlation between anaemia and BMI. Furthermore, Gargade and Patil^(87)^ found similar findings of the higher prevalence of anaemia among normal weight 55⋅2 %, underweight 27⋅6 %, overweight 13⋅6 % and obese 3⋅4 %. Metha^(88)^ also found that anaemia is more prevalent 63⋅33 % among underweight student and overweight students have the less prevalence of anaemia (0⋅83 %), while the prevalence of anaemia was 6⋅67 % in normal weight students. High Hb content recorded for overweight participants may probably be due to the nutritional status of these groups such as intake of high iron foods and also due to over-nutrition. The study done by Khan *et al.*^(42,43)^ also showed a statistically significant positive correlation of BMI with haemoglobin. Contrary to this, Abro *et al.*^(45)^ also found a negative correlation between Hb content and BMI among adolescents.

Despite the fact that overweight or obesity has been reported to induce inflammatory problems through release of hepcidine, an inhibitor of dietary iron absorption which may cause anaemia among overweight or obese individuals^(30,45,89)^, the percentages of anaemia among participants with high BMI were quite less. This may probably be due to the reasons majority of the overweight participants were having active lifestyles in an attempt to shed their extra weight and were on dietary intake of camel milk which contains greater iron concentration (1⋅35–2⋅5 mg/l *v.* 0⋅3–0⋅8 mg/l)^(90)^. Since the majority of iron in camel's milk is associated with the lower molecular fraction of casein suggesting better bioavailability to increase iron store and haemoglobin synthesis^(91)^, this might probably be the reason that the majority of overweight and obese participants could not develop anaemia. Furthermore, vitamin C concentration is also higher in camel milk^(92,93)^ which enhances iron absorption from non-heme sources by reducing ferric iron into the readily bioavailable form – ferrous iron. These characteristics might have been beneficial for better haemoglobin synthesis.

Another reason may be that the participants were non-vegetarian in their food preferences and prefer camel meat, traditional seafood and organ meats which are rich in the bioavailable form of iron. In addition, these animal foods are good source of high-quality proteins with the capacity to enhance iron absorption from non-heme sources consequently increasing blood haemoglobin concentrations^(94–96)^ thus diluting the adverse effects of overweight on Hb synthesis.

Moreover, the percentage of anaemia in the present study was 51⋅6 % which is lower than that reported from several low-income countries, such as India^(80)^, Ethiopia^(97–99)^, Nepal^(100)^, Brazil^(101)^ and Latin America^(77)^ however, is almost similar to that reported from high-income countries like Canada^(102)^ and the United States of America^(103)^.

The percentages of moderate and severe anaemia in the present study were comparatively low at around 15⋅4 and 7⋅69 %, respectively. Such lesser prevalence of severe anaemia seems logical and could be justified by several factors. First, citizens have a high standard of living, which includes free education and medical care in addition to subsidised foods, such as iron-fortified bread and iron-fortified food whereas these factors remain strong predictors for anaemia in various other low- and middle-income countries^(104)^. Second, the study participants had high hygienic practices which completely rules out the chances and occurrence of anaemia due to parasites or infections. Percentages of mild anaemia recorded in the present study are much lesser than that recorded for the female students of Faculty of Applied Medical Sciences in Jazan University (67⋅35 %)^(44)^.

In the present study, there was no significant relation between anaemia and family education as analyses of questionnaire revealed that family members mainly parents of all the study participants were educated with some were being bilingual as Arabic and English (Table 1). The present study shows a statistically insignificant association of anaemia and socio-economic status (*P* > 0⋅05). Similar results are found in a study done by Ugwuja *et al.*^(53)^, which has also depicted that educational status and occupation had no effect on anaemia prevalence.

Though the incidence of moderate and severe anaemia is only 15⋅4 and 7⋅69 %, respectively, in the present study, it is still disheartening, as these students have better knowledge about anaemia and its consequences, compared to general population. The contributing factors could be the stress of the professional course which demands comparatively longer study hours, night shifts in hospitals during field trainings and changes in the dietary habits in most of the students, as they enter university.

## Conclusion

To sum up, we may say that the percentage of mild anaemia are high among female nursing undergraduates of Farasan which in long term may affect cognitive, learning and work efficiencies of these future nurses. It is a matter of concern as this may probably be due to negligence of healthy food habits as majorities were found to skip their daily breakfast which is very important for a normal kick of the metabolism and, also due to more preference to junk foods.

The present study is a significant contribution in highlighting the problem of anaemia, and its association with breakfast skipping and BMI among educated youth. The outcomes of the study would also be useful in healthcare planning and to make policies to strategically reduce the incidence of anaemia.

## Recommendations

Young girls of age 18–25 years are more prone to get nutritional anaemia. Routine checkup and haemoglobin estimation should be done frequently for the screening of anaemia as the majority of the participants were not even aware of that they are anaemic. Preventive programmes and policies should be made to combat this health issue.

Haemoglobin estimation of students at the time of students’ registration should be done. Iron and folic acid tablets in therapeutic doses should be provided to anaemic students by college clinic and expert general physician should be appointed in college clinic so that they could have appropriate medications. Also, a dietician should be appointed in the clinic so that the students specifically those who are staying far from their families could be motivated and educated to take balanced diet with a regular and healthy breakfast because nutritional anaemia is totally preventable.
